# Discriminating Alzheimer's Pathologic Changes from Dementias: A Multi‐Factor Blood Biomarkers Diagnostic Model Using the AT(N) System

**DOI:** 10.1002/alz.090706

**Published:** 2025-01-09

**Authors:** Tianyi Wang, Li Shang, Chenhui Mao, Longze Sha, Liling Dong, Caiyan Liu, Dan Lei, Jie Li, Jie Wang, Xinying Huang, Bo Li, Yixuan Huang, Jialu Bao, Yuyue Qiu, Shanshan Chu, Wei Jin, Zhaohui Zhu, Huimin Sui, Bo Hou, Feng Feng, Bin Peng, Liying Cui, Jianyong Wang, Qi Xu, Jing Gao

**Affiliations:** ^1^ Peking Union Medical College Hospital, Beijing China; ^2^ Institute of Basic Medical Sciences & Neuroscience Center, Chinese Academy of Medical Sciences and Peking Union Medical College, Beijing China; ^3^ Tsinghua University, Beijing China

## Abstract

**Background:**

To assess the diagnostic efficacy of plasma biomarkers in distinguishing Alzheimer's pathologic changes from other forms of dementia, our study focused on examining plasma biomarkers in accordance with the AT(N) system.

**Method:**

A total of 156 participants were recruited from the PUMCH dementia cohort. This comprised 55 non‐AD (A‐) and 101 Alzheimer's pathologic changes (A+) individuals. Among the latter group, there were 40 AD‐like(A+T‐) and 61 AD(A+T+). Biomarker diagnoses were relied on cerebrospinal fluid (CSF) biomarkers and amyloid PET results. Plasma biomarkers were quantified using single molecule array (SIMOA).

**Result:**

Plasma Aβ40, Aβ42, GFAP, NfL, and pTau181 were compared among A‐, A+T‐, and A+T+ groups. Significant reductions in plasma Aβ42/40, along with elevated levels of plasma pTau181 and GFAP, were observed in both A+T‐ and A+T+ groups compared to the A‐ group. However, no significant differences were found between the A+T‐ and A+T+ groups. Additionally, plasma Aβ42/40, pTau181, and GFAP exhibited significant correlations with multiple CSF biomarkers, especially with CSF Aβ biomarkers, whereas plasma Aβ42, Aβ40, and NfL did not. None of the plasma biomarkers were indicative of disease severity in A+ dementia patients. Finally, a high accuracy diagnostic model in predicting Alzheimer's pathologic changes in dementia patients could be generated using plasma biomarkers.

**Conclusion:**

Plasma levels of Aβ42/40, pTau181, and GFAP present valuable indicators in distinguishing Alzheimer's pathologic changes from other forms of dementia. The integration of multiple plasma biomarkers into a diagnostic model can significantly enhance diagnostic accuracy, thereby providing valuable support for clinical practice.

